# Supplementary material for: Transcriptional profiling of matched patient biopsies clarifies molecular determinants of enzalutamide-induced lineage plasticity
Source: Nat Commun. 2022 Sep 15;13:5345. doi: 10.1038/s41467-022-32701-6 (PMC9477876; doi:10.1038/s41467-022-32701-6)
Supplement: Supplementary file 1 — Supplementary Information [file 41467_2022_32701_MOESM1_ESM.pdf]

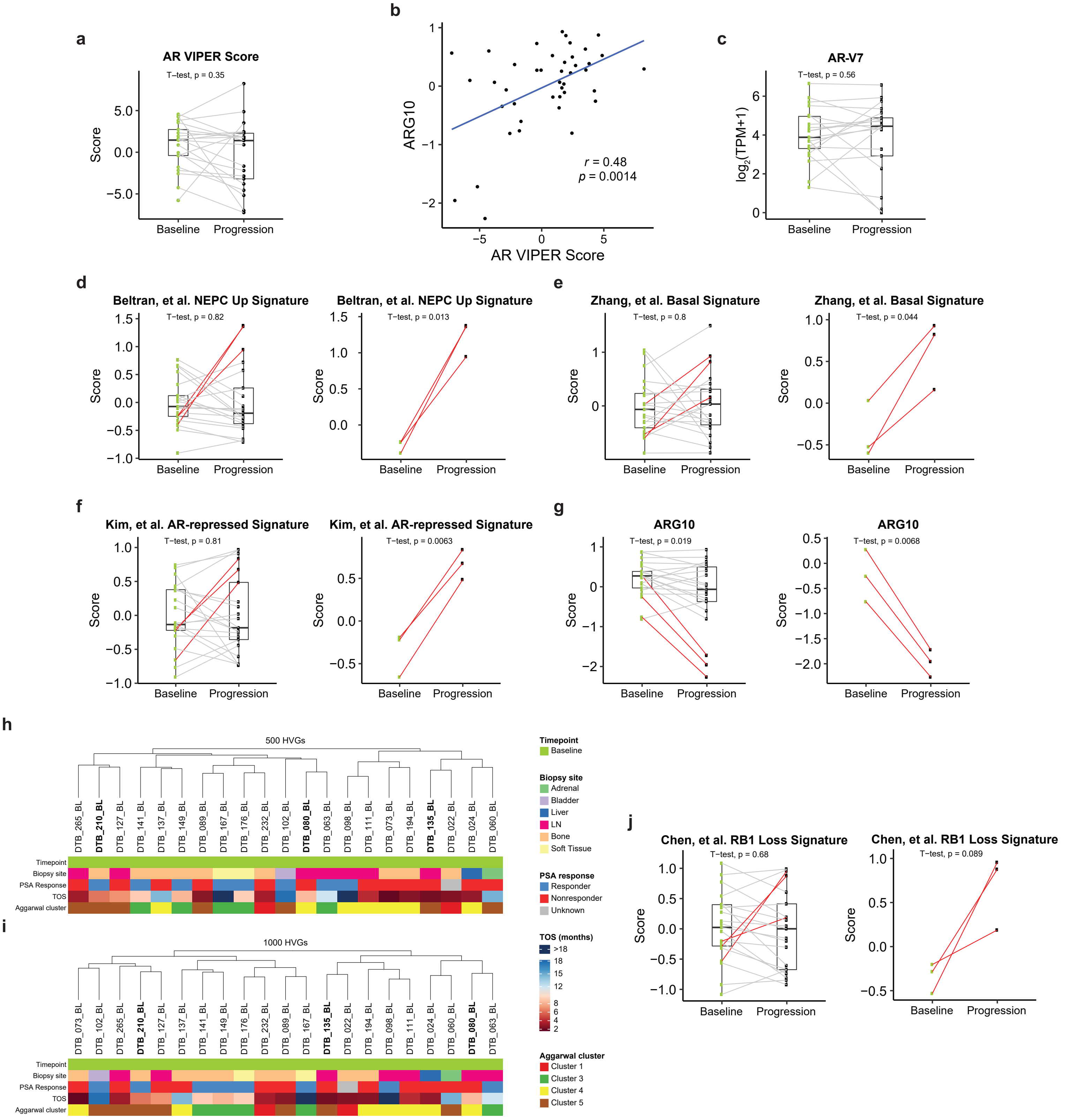

Supplementary Figure 1: a AR VIPER Score for each baseline and progression sample. **b** ARG10 and VIPER AR score are strongly correlated. p-value shown was determined using a two-tailed Pearson correlation. **c** AR-V7 splice variant expression for each baseline and progression sample. **d-g** Signature scores were calculated for baseline and progression samples using Beltran, et al. NEPC upregulated genes[5] in **d**, Zhang, et al. basal genes[4] in **e**, Kim, et al. AR-repressed lineage plasticity genes[7] in **f**, and ARG10 genes[6] in **g**. Red lines link converter baseline and progression samples. All 42 baseline and progression samples were used in **a-g**. **h** Unsupervised hierarchical clustering of all baseline samples (n=21) using top 500 differentially expressed genes. **i** Unsupervised hierarchical clustering of all baseline samples (n=21) using top 1000 differentially expressed genes. **j** Signature scores were calculated for baseline and progression samples (n=42) using genes upregulated with RB1 loss described by Chen, et al.[8] For all boxplots, the center line represents the median; the box represents the interquartile range (IQR; the range between the 25th and 75th percentile); and the whiskers represent 1.58 × IQR. For **a**, **c-g**, and **j**, p-value shown was determined using a two-tailed paired Student's t-test. Source data are provided as a Source Data file.

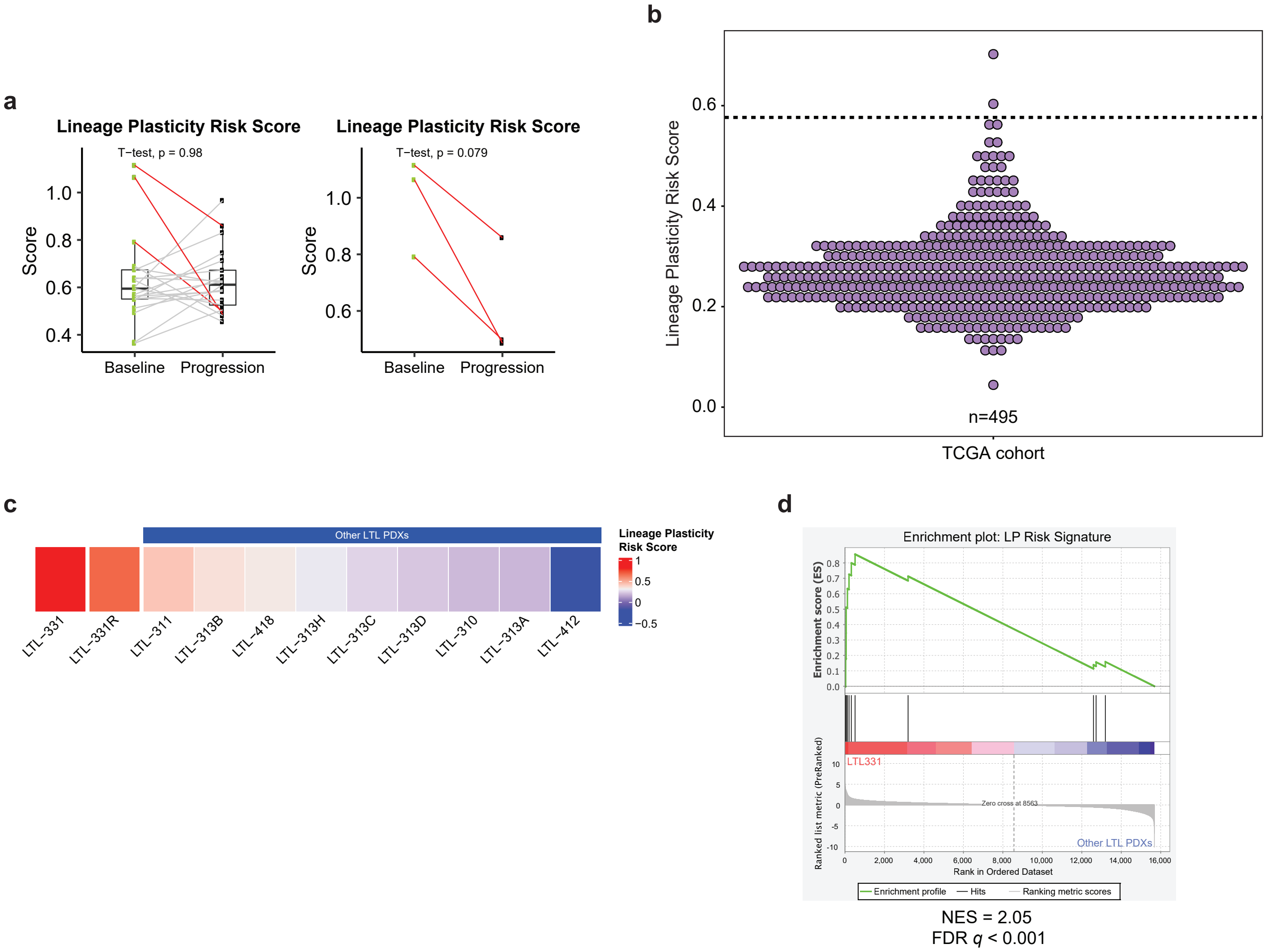

Supplementary Figure 2: a Lineage plasticity risk scores were calculated for baseline vs. progression samples (n=42). Red lines link converter baseline and progression samples. For the boxplot, the center line represents the median; the box represents the interquartile range (IQR; the range between the 25th and 75th percentile); and the whiskers represent 1.58 × IQR. p-value shown was determined using a two-tailed paired Student's t-test. **b** Dotplot showing lineage plasticity risk signature score for patients described in prostate cancer TCGA[15]. **c** Heatmap showing lineage plasticity risk score in LTL331, other hormone-naïve LTL PDXs, and LTL331R described by Lin, et al.[11] **d** Gene set enrichment plot for 14 gene lineage plasticity risk signature in LTL331 vs. other nine hormone-naïve LTL PDXs described in Lin, et al.[11] Source data are provided as a Source Data file.

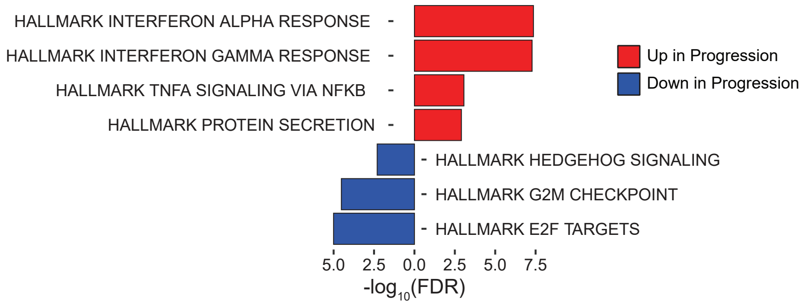

Supplementary Figure 3: Hallmark pathway analysis demonstrating the top up- or downregulated pathways in progression vs. baseline samples for the 18 patients whose tumors did not convert. Source data are provided as a Source Data file.

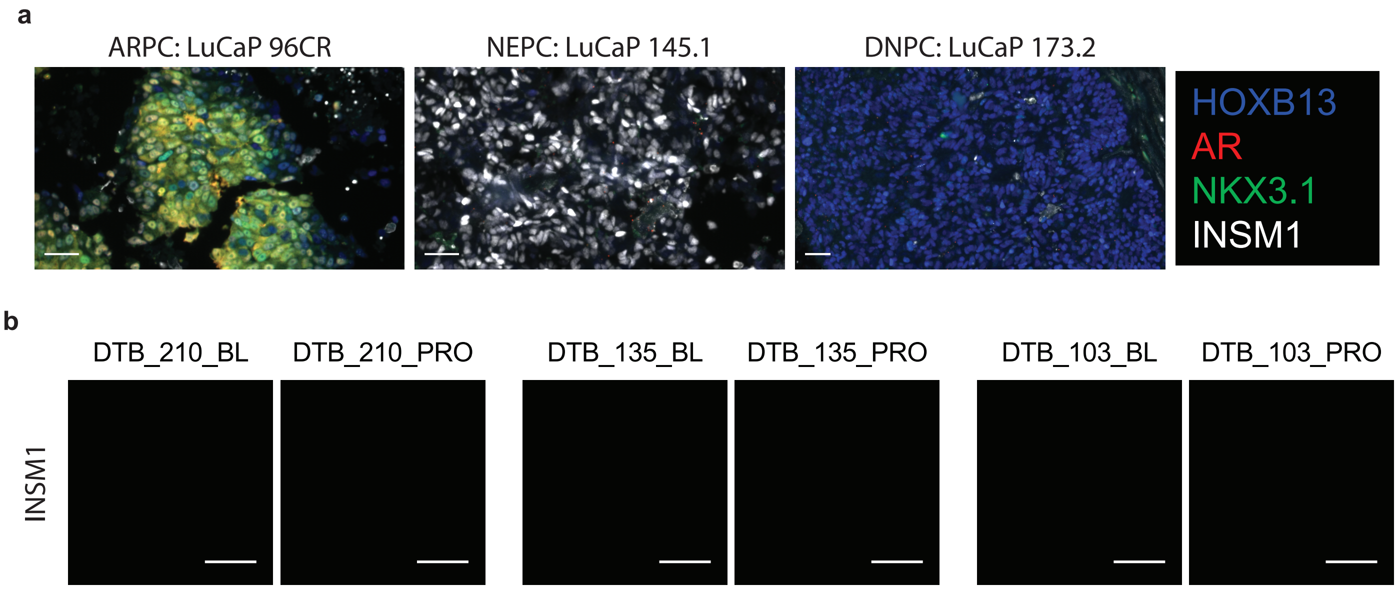

Supplementary Figure 4: a Panels show expression of AR, NKX3.1, INSM1, and HOXB13 in ARPC LuCaP 96CR PDX tumor, NEPC LuCaP 145.1 PDX tumor, and DNPC LuCaP 173.2 PDX tumor. AR and NKX3.1 were only expressed in LuCaP 96CR. INSM1 was only expressed in LuCaP 145.1, while HOXB13 was expressed in both LuCaP 96CR and LuCaP 173.2. Scale bars represent 50μm. **b** Absent INSM1 expression in all three matched converter samples examined. Scale bars represent 50μm. For **a-b**, single replicates were performed for all samples.

Supplementary Table 1: Patient demographics and clinical information summary

Patients		n=21
Median age at time of enrollment (range)		71 (58-88)
Gleason score at diagnosis		
≥8		16
<8		5
ECOG performance status		
0		10
1		11
Metastatic site biopsied baseline (progression)		
	Bone	9 (9)
	Lymph node	7 (8)
	Pelvic soft tissue	2 (1)
	Bladder wall	1 (0)
	Liver	1 (2)
	Adrenal	1 (1)
	Same lesion biopsied	8
Visceral metastatic disease at time of biopsy		5
Prior treatment		
	Abiraterone	7
	ADT	21
	Bicalutamide	8
	Cabazitaxel	1
	Docetaxel	3
	Sip-T	1
Median PSA at enrollment (range)		57 (3.6-1210)
50% PSA response to enzalutamide		7
Median time on enzalutamide, days (SD)		226 (207)

Supplementary Table 2: Patient and biopsy information

Sample_ID	Baseline biopsy tissue	Progression biopsy tissue	Same site biopsied	Time Between Biopsies (days)	PSA at baseline (ng/mL)	PSA Change at 12 weeks (%)	Prior treatment
DTB_022	Bone	Bone	No	85	7.28	N/A	ADT, abiraterone
DTB_024	Liver	Liver	No	99	48.92	75.05	ADT, abiraterone, docetaxel
DTB_060	Adrenal	Adrenal	Yes	449	102.45	-20.41	ADT
DTB_063	LN	LN	No	368	20.9	-74.64	ADT
DTB_073	Bone	Bone	No	54	57.67	156.81	ADT, abiraterone
DTB_080	LN	LN	Yes	266	14.92	-28.48	ADT
DTB_089	Bone	Liver	No	91	14.27	181.18	ADT, abiraterone
DTB_098	LN	LN	Yes	615	148.39	-83.74	ADT
DTB_102	Bladder	LN	No	533	1210.48	-93.02	ADT, abiraterone, docetaxel, cabazitaxel
DTB_111	LN	LN	Yes	134	35.02	113.99	ADT, abiraterone
DTB_127	LN	LN	Yes	226	228	169.29	ADT, abiraterone
DTB_135	LN	LN	No	73	9.19	164.26	ADT, bicalutamide
DTB_137	Bone	Bone	No	441	539.62	-10.85	ADT, bicalutamide
DTB_141	Bone	Bone	No	285	140.07	-81.04	ADT, bicalutamide
DTB_149	Bone	Bone	No	262	16.45	-80.29	ADT, bicalutamide
DTB_167	Soft tissue	Bone	No	827	136.64	-88.38	ADT, bicalutamide, sipuleucel-T
DTB_176	Soft tissue	Soft tissue	Yes	291	3.57	-64.76	ADT, bicalutamide
DTB_194	Bone	Bone	No	88	103.55	100.36	ADT, bicalutamide
DTB_210	Bone	Bone	No	200	70.45	-83.36	ADT
DTB_232	Bone	Bone	Yes	114	5.31	-0.55	ADT, docetaxel
DTB_265	LN	LN	Yes	105	42.93	5.84	ADT, bicalutamide

Supplementary Table 3: 14 gene Lineage Plasticity Risk Signature

The Wald test implemented in DESeq2 R package was used to calculate p-values.

	log2FoldChange	p-value	p-adj
RNF43	3.119769769	3.48E-07	0.001120635
SNRPF	2.552743092	2.82E-06	0.005047666
TRABD2A	4.179997895	4.15E-06	0.006073226
NDUFA12	2.140173456	5.32E-06	0.00659587
GAS2L3	2.638572495	8.07E-06	0.008662214
RPS24	1.711754964	1.67E-05	0.012808225
DNA2	1.418005136	1.91E-05	0.014009937
RP5-857K21.10	1.92284875	0.000294085	0.098659293
POC1B	1.891507067	3.43E-05	0.023013467
ADK	1.506396028	0.000276788	0.098322909
ATP5B	1.298983588	0.000195146	0.080498034
XPOT	1.037507837	0.000166796	0.075841951
SLCO1B3	5.897966677	0.000174263	0.075841951
RHOBTB1	2.882280784	0.000112042	0.05976354

Supplementary Table 4: High converter risk score TP53/RB1/PTEN alterations

Dataset	Sample ID	RB1	TP53	PTEN
Abida	PRAD-01115468-Tumor-SM-6B2KE	Frameshift deletion	None	Frameshift deletion
Abida	PRAD-01115554-Tumor-SM-A56E4	None	Missense	None
Abida	PRAD-6115594-0-Tumor-SM-B2XRW	None	None	None
Abida	PROS01448-1115161-Tumor-SM-5SGU1	None	None	None
Abida	SC-9146-T	None	None	None
Abida	MO-1337-TM	None	Deep deletion	Deep deletion
Alumkal	251	Not available	Not available	Not available
Alumkal	206	Not available	Not available	Not available
Alumkal	229	None	None	None

Supplementary Table 5: DNA alterations for wider cohort

Sample_ID	Mutation	Copy number gain/loss
DTB_022_BL	Inadequate sample	Inadequate sample
DTB_022_Pro	BRAF	None found
DTB_024_BL	Inadequate sample	Inadequate sample
DTB_024_Pro	PTEN	None found
DTB_060_BL	None found	None found
DTB_060_Pro	None found	None found
DTB_063_BL	AKT1, HRAS, TP53	None found
DTB_063_Pro	AKT1, HRAS, TP53	None found
DTB_073_BL	TP53	None found
DTB_073_Pro	TP53	None found
DTB_080_BL	PTEN	None found
DTB_080_Pro	PTEN	None found
DTB_089_BL	None found	None found
DTB_089_Pro	TP53	None found
DTB_098_BL	None found	None found
DTB_098_Pro	ATM, NTRK1	ATM loss, MYC amplification
DTB_102_BL	TP53	None found
DTB_102_Pro	TP53, NF1	TP53 loss
DTB_103_BL	RB1, FGFR3, NOTCH1	None found
DTB_103_Pro	RB1, FGFR3, NOTCH1	None found
DTB_111_BL	KDR, TP53	CDKN2A loss
DTB_111_Pro	KDR, TP53	CDKN2A loss
DTB_127_BL	TP53, TSC2	RB1 loss
DTB_127_Pro	TP53, TSC2	RB1 loss
DTB_135_BL*	SPEN, FAT1	AR amplification, MYC amplification
DTB_135_Pro*	SPEN, FAT1, CTNNB1 (subclonal)	AR amplification, MYC amplification
DTB_137_BL	Inadequate sample	Inadequate sample
DTB_137_Pro	NOTCH1	None found
DTB_141_BL	NF1	PTEN loss
DTB_141_Pro	NF1	PTEN loss, TP53 loss
DTB_149_BL	PTEN, PIK3R1, STK11	None found
DTB_149_Pro	PTEN, PIK3R1, STK11	TP53 loss
DTB_167_BL	PTEN, ERBB2	FGFR1 loss
DTB_167_Pro	PTEN, XRCC1	AR amplification
DTB_176_BL	PTEN	PTEN loss, TP53 loss
DTB_176_Pro	Inadequate sample	Inadequate sample
DTB_194_BL	None found	None found
DTB_194_Pro	None found	None found
DTB_210_BL*	APC, SPOP, KMT2C	None Found
DTB_210_Pro*	APC, SPOP, KMT2C	None Found
DTB_232_BL	None found	None found
DTB_232_Pro	None found	None found
DTB_265_BL	ARID1A, FANCC, FANCM, POLE, TP53	AR amplification
DTB_265_Pro	ARID1A, ERBB4, FANCC, FANCM, MAP2K2, POLE, TP53	AR amplification, Chromosome 8 amplification

*Tested via cfDNA assay

Supplementary Table 6: Gene signature composition

Beltran, et al. NEPC Up	Zhang, et al. Basal	Kim, et al. AR-repressed	ARG10	Chen, et al. Up with RB1 loss
ASXL3	COL17A1	NRXN3	ALDH1A3	RIBC2
AURKA	CSMD2	ALX4	KLK3	SH3GL2
BRINP1	CDH13	TRAF3IP2	FKBP5	IQCC
C7orf76	MUM1L1	ATP2C2	KLK2	MESP1
CAND2	MMP3	KDM4A	NKX3-1	CAMK2N2
DNMT1	IL33	TGFBR3	TMPRSS2	DONSON
ETV5	GIMAP8	SEMA3C	PLPP1	USP1
EZH2	PDPN	RBL1	PART1	POU4F1
GNAO1	VSNL1	MET	PMEPA1	LHX2
GPX2	BNC1	CIT	STEAP4	CDKN2A
JAKMIP2	IGFBP7	CHAC1		ZSCAN16
KCNB2	DLK2	CABLES1		GPR137C
KCND2	HMGA2	FLNB		H2AFV
KIAA0408	NOTCH4	DAB2IP		AKAP5
LRRC16B	THBS2	AUTS2		ELF2
MAP10	TAGLN	DAB1		CDKAL1
MYCN	FHL1	CDC42EP4		ZNF43
NRSN1	ANXA8L2	CD55		TXNDC16
PCSK1	COL4A6	TTLL3		ZNF606
PROX1	KCNQ5	RP11-159F24.1		PCSK1
RGS7	WNT7A	MYO15B		RAD1
SCG3	KCNMA1	BCLAF1		CCNE2
SEC11C	NIPAL4	RIMS1		STMN1
SEZ6	FLRT2	NEFL		ZNF254
SOGA3	LTBP2	GPD2		ID4
ST8SIA3	FOXI1	HPCAL4		ZSCAN18
SVOP	NGFR	SCRN1		CDKN2C
SYT11	SERPINB13	TACC2		HSD17B6
TRIM9	CNTNAP3B	APBB2		PDIK1L
	FGFR3	CDCA7L		FANCL
	ARHGAP25	GABRA5		TEX9
	AEBP1	MGST1		COCH
	FJX1	DPF1		PIK3R3
	TNC	RAI14		RNASEH2A
	MSRB3	PARP12		ACYP1
	NRG1	PLXNA2		UBR7
	SERPINF1	EPB41L2		CLSPN
	DLC1	IGSF9B		BTF3L4
	IL1A	RCOR1		KCND2
	DKK3	SMAD7		TIGD7
	ERG	MAP2K6		ZNF528
	SYNE1	FHOD3		DAND5
	JAG2	BIN1		TTLL7
	JAM3	TMOD1		DCHS1
	MRC2	SMAD6		ZNF367
	SPARC	DUSP5		GLRB
	C16orf74	HUNK		RTN3
	FAT3	MYO10		ZNF347
	KIRREL	CXorf57		EID2B
	SH2D5	SMC6		GKAP1
	KRT6A	ARHGEF3		ZNF610
	KRT34	STRBP		UCHL1
	ITGA6	STXBP6		KIAA1841
	TP63	ROBO1		KLHL2
	KRT5	TANC2		JAKMIP1
	KRT14	FRMD3		SLC36A4
		GOLIM4		FAM111B
		DPP10		CPE
		WSCD1		NEURL1B
		TNFAIP2		MNS1
		EPHA6		TCTEX1D2
		SH3GL2		SPSB4
		BCL2		KCTD16
		BEND3		CAND2
		MBP		OSCP1
		SAMD5		DEK
		TMEM65		FBLN7
		MYB		ZBTB8A
		ASXL2		
		HRH2		
		KIAA0319		
		CREB5		
		AK5		
		PALM2-AKAP2		
		IKZF3		
		ARHGEF28		

Supplementary Methods

Aggarwal, et al. cluster designation

The unsupervised analysis from Aggarwal, et al.[1] identified five clusters using 119 samples. That study identified 528 genes that were the most differentially expressed between the clusters. Using that gene list, we determined the cluster assignments for new samples included in this matched biopsy cohort without replicating the unsupervised analysis. First, we addressed the sample batch effect between the samples from the previous study and those from the current study. We applied exponential normalization on the expression data of all samples—old and new. Exponential normalization is a per-sample operation that fits the expression of all genes to a unit exponential distribution. Next, we used scikit-learn's k-nearest-neighbor classifier implementation[2] to train a classification model using 118 exponential-normalized samples that had pre-existing cluster assignments. The model used 507 genes from the 528 gene list from Aggarwal, et al.[1] because several genes were not expressed in the previously uncharacterized samples used in this report. The model's accuracy in leave-one-out cross validation was 0.712. The trained model was then used to predict the cluster assignment of previously unclassified, exponential-normalized samples.

Labrecque, et al. classification

To determine the Labrecque classification, we applied a 26 gene signature used previously to define five phenotypic categories of CRPC[3]: AR-high prostate cancer (ARPC), amphicrine prostate cancer, AR-low prostate cancer (ARLPC), double-negative prostate cancer (DNPC), and neuroendocrine prostate cancer (NEPC)[3]. One gene (TARP) was missing from the dataset and was not included. Samples were assigned to the phenotype groups by clustering using Euclidean distance calculated by the dist function and visualization using classical

multidimensional scaling (MDS) calculated with the cmdscale function in R using the $\log_2$(TPM + 1) transformed expression profiles of the remaining 25 genes.

Single-sample gene signature scores

In this study, we used several gene signatures collected from public resources, including the Zhang Basal gene signature[4]; the Beltran, et al. NEPC Up gene signature[5]; the ARG10 signature[6]; the Kim, et al. 76 gene AR-repressed signature[7]; and the Chen, et al. RB1 loss signature[8]. The signature genes are listed in Supplementary Table 6. TPM gene expression values were $\log_2$(TPM + 1) transformed and converted to z-scores by: $z = (x - \mu)/\sigma$, where μ is the average $\log_2$(TPM + 1) across all samples of a gene and σ is the standard deviation of the $\log_2$(TPM + 1) across all samples of a gene. The signature score of each sample was the average z-score of all genes in each signature. p-values were determined using a two-tailed paired Student's t-test.

Development of a lineage plasticity risk gene signature

To derive the lineage plasticity risk signature, differential gene expression analysis was performed using DESeq2 as described above by comparing baseline converter vs. non-converter samples. Genes upregulated in converter samples with adjusted p-value < 0.1 were included. Single-sample lineage plasticity risk signature was derived using the single-sample gene set enrichment analysis (ssGSEA)[9] implemented in the GSVA[10] R package.

Assessment of the lineage plasticity signature in patient-derived xenograft models

We examined baseline gene expression from 11 human prostate PDX models[11]. We compared the lineage plasticity signature scores of the one tumor (LTL331) that undergoes castration-induced lineage plasticity to other hormone-naïve PDXs that do not: LTL310, LTL311, LTL412, LTL418, LTL313A, LTL313B, LTL313C, LTL313D, LTL313H and the

castration-resistant LTL331R PDX. The single-sample lineage convert signature score was derived using the single-sample gene set enrichment analysis (ssGSEA)[9] implemented in the GSVA[10] R package. The fold-change-based gene ranking from the comparison excluding LTL331R was used to assess the enrichment of the lineage plasticity risk signature using gene set enrichment analysis[12].

Survival analysis

We evaluated the correlation of the lineage plasticity risk signature with survival time in two independent datasets. First, after excluding patients that overlapped with this current study, we identified 17 patients whose tumors had undergone RNA-seq from our prior prospective enza clinical trial with overall survival information[13]. Second, we identified samples from the International Dream Team dataset for which overall survival from first line ARSI treatment was available; we restricted the patients to those without prior exposure to abiraterone, enza, or docetaxel[14]. Then, we merged the gene expression of the three datasets, including the samples in the matched biopsy cohort, into one matrix to calculate the enrichment score of each sample consistently. Single-sample lineage plasticity risk score was derived using the single-sample gene set enrichment analysis (ssGSEA)[9] implemented in the GSVA[10] R package. We then defined a signature cutoff to separate the baseline converter samples from the non-converter samples from the matched biopsy cohort with the maximum margin as calculated by taking the average of the lowest score in the non-convert group and highest score in the converter group. Finally, we used this cutoff to stratify samples in the two independent datasets into two groups with high and low lineage plasticity signature risk scores. The comparison of the survival pattern between the two groups was performed by the Kaplan-Meier method using the Mantel-Cox log-rank test. Separately, we applied the same cutoff defined above to stratify samples from the prostate cancer Cancer Genome Atlas[15].

SU2C sample relabeling

For several samples, SU2C IDs were relabeled as baseline or progression based upon when the patient was exposed to enzalutamide. DTB_022_PRO and DTB_024_PRO were relabeled DTB_022_BL and DTB_024_BL, respectively, as those biopsies were performed immediately prior to starting enzalutamide treatment. Correspondingly, DTB_022_PRO2 and DTB_024_PRO2 were relabeled DTB_022_PRO and DTB_024_PRO as those biopsies were performed at progression on enzalutamide. DTB_098_PRO2 was relabeled DTB_098_PRO as patient continued enzalutamide until after PRO2 biopsy.

Supplementary References

1. Aggarwal, R., *et al.* Clinical and Genomic Characterization of Treatment-Emergent Small-Cell Neuroendocrine Prostate Cancer: A Multi-institutional Prospective Study. *J Clin Oncol* **36**, 2492-2503 (2018).
2. Pedregosa, F., *et al.* Scikit-learn: Machine Learning in Python. *J Mach Learn Res* **12**, 2825-2830 (2011).
3. Labrecque, M.P., *et al.* Molecular profiling stratifies diverse phenotypes of treatment-refractory metastatic castration-resistant prostate cancer. *J Clin Invest* **129**, 4492-4505 (2019).
4. Zhang, D., *et al.* Stem cell and neurogenic gene-expression profiles link prostate basal cells to aggressive prostate cancer. *Nat Commun* **7**, 10798 (2016).
5. Beltran, H., *et al.* Divergent clonal evolution of castration-resistant neuroendocrine prostate cancer. *Nat Med* **22**, 298-305 (2016).
6. Nyquist, M.D., *et al.* Combined TP53 and RB1 Loss Promotes Prostate Cancer Resistance to a Spectrum of Therapeutics and Confers Vulnerability to Replication Stress. *Cell Rep* **31**, 107669 (2020).
7. Kim, D.H., *et al.* BET Bromodomain Inhibition Blocks an AR-Repressed, E2F1-Activated Treatment-Emergent Neuroendocrine Prostate Cancer Lineage Plasticity Program. *Clin Cancer Res* **27**, 4923-4936 (2021).
8. Chen, W.S., *et al.* Genomic Drivers of Poor Prognosis and Enzalutamide Resistance in Metastatic Castration-resistant Prostate Cancer. *Eur Urol* **76**, 562-571 (2019).
9. Barbie, D.A., *et al.* Systematic RNA interference reveals that oncogenic KRAS-driven cancers require TBK1. *Nature* **462**, 108-112 (2009).
10. Hanzelmann, S., Castelo, R. & Guinney, J. GSVA: gene set variation analysis for microarray and RNA-seq data. *BMC Bioinformatics* **14**, 7 (2013).
11. Lin, D., *et al.* High fidelity patient-derived xenografts for accelerating prostate cancer discovery and drug development. *Cancer Res* **74**, 1272-1283 (2014).
12. Subramanian, A., *et al.* Gene set enrichment analysis: a knowledge-based approach for interpreting genome-wide expression profiles. *Proc Natl Acad Sci U S A* **102**, 15545-15550 (2005).
13. Alumkal, J.J., *et al.* Transcriptional profiling identifies an androgen receptor activity-low, stemness program associated with enzalutamide resistance. *Proc Natl Acad Sci U S A* **117**, 12315-12323 (2020).
14. Abida, W., *et al.* Genomic correlates of clinical outcome in advanced prostate cancer. *Proc Natl Acad Sci U S A* **116**, 11428-11436 (2019).
15. Cancer Genome Atlas Research Network. The Molecular Taxonomy of Primary Prostate Cancer. *Cell* **163**, 1011-1025 (2015).
