# Supplementary material for: Transcriptional profiling of matched patient biopsies clarifies molecular determinants of enzalutamide-induced lineage plasticity
Source: Nat Commun. 2022 Sep 15;13:5345. doi: 10.1038/s41467-022-32701-6 (PMC9477876; doi:10.1038/s41467-022-32701-6)
Supplement: Supplementary file 3 — Reporting Summary [file 41467_2022_32701_MOESM3_ESM.pdf]

nature portfolio

Corresponding author(s): Joshi Alumkal

Last updated by author(s): Jul 26, 2022

Reporting Summary

Nature Portfolio wishes to improve the reproducibility of the work that we publish. This form provides structure for consistency and transparency in reporting. For further information on Nature Portfolio policies, see our Editorial Policies and the Editorial Policy Checklist.

Statistics

For all statistical analyses, confirm that the following items are present in the figure legend, table legend, main text, or Methods section.

n/a	Confirmed	
☐	☒	The exact sample size (*n*) for each experimental group/condition, given as a discrete number and unit of measurement
☐	☒	A statement on whether measurements were taken from distinct samples or whether the same sample was measured repeatedly
☐	☒	The statistical test(s) used AND whether they are one- or two-sided<br>*Only common tests should be described solely by name; describe more complex techniques in the Methods section.*
☒	☐	A description of all covariates tested
☐	☒	A description of any assumptions or corrections, such as tests of normality and adjustment for multiple comparisons
☐	☒	A full description of the statistical parameters including central tendency (e.g. means) or other basic estimates (e.g. regression coefficient) AND variation (e.g. standard deviation) or associated estimates of uncertainty (e.g. confidence intervals)
☐	☒	For null hypothesis testing, the test statistic (e.g. *F*, *t*, *r*) with confidence intervals, effect sizes, degrees of freedom and *P* value noted<br>*Give P values as exact values whenever suitable.*
☒	☐	For Bayesian analysis, information on the choice of priors and Markov chain Monte Carlo settings
☒	☐	For hierarchical and complex designs, identification of the appropriate level for tests and full reporting of outcomes
☒	☐	Estimates of effect sizes (e.g. Cohen's *d*, Pearson's *r*), indicating how they were calculated

Our web collection on statistics for biologists contains articles on many of the points above.

Software and code

Policy information about availability of computer code

Data collection	No software was used for data collection.
Data analysis	R version (version 3.5.1), DESeq2 R package (version 1.22.2), limma R package (version 3.38.3), viper R package (version 1.16.0), GSVA (version 1.42.0), Python (version 3.8.11), sklearn (version 0.24.1), FastQC (version 0.11.8), RSEM 50 (version 1.3.1), QuPath (version 0.3.0), MsigDB (version 7.0), ichorCNA package in R (version 0.3.2), GraphPad Prism (version 9.3.1). All code used in this study is publicly available.

For manuscripts utilizing custom algorithms or software that are central to the research but not yet described in published literature, software must be made available to editors and reviewers. We strongly encourage code deposition in a community repository (e.g. GitHub). See the Nature Portfolio guidelines for submitting code & software for further information.

Data

Policy information about availability of data

All manuscripts must include a data availability statement. This statement should provide the following information, where applicable:

- Accession codes, unique identifiers, or web links for publicly available datasets
- A description of any restrictions on data availability
- For clinical datasets or third party data, please ensure that the statement adheres to our policy

The raw RNA-seq data generated in this study have been deposited in the EGA database under Study ID EGAS00001005954 [https://ega-archive.org/studies/EGAS00001005954]. Additionally, the RNA-seq data (TPM) generated in this study are provided in a Supplementary Data file.

Field-specific reporting

Please select the one below that is the best fit for your research. If you are not sure, read the appropriate sections before making your selection.

☒ Life sciences ☐ Behavioural & social sciences ☐ Ecological, evolutionary & environmental sciences

For a reference copy of the document with all sections, see nature.com/documents/nr-reporting-summary-flat.pdf

Life sciences study design

All studies must disclose on these points even when the disclosure is negative.

Sample size	No statistical method was used to predetermine sample size. All patients from the Stand Up to Cancer Foundation/Prostate Cancer Foundation West Coast Dream Team who underwent a metastatic tumor biopsy prior to enza and a repeat biopsy at the time of progression and whose tumor cells underwent RNA-sequencing after laser capture microdissection were included.
Data exclusions	No data were excluded from the analyses.
Replication	One sample per biopsy was sequenced. Only one biopsy sample was obtained from each patient at each timepoint; therefore, only sample was sequenced per timepoint with no replicates.
Randomization	The experiments were not randomized. This was a longitudinal cohort study without randomization.
Blinding	The Investigators were not blinded to allocation during experiments and outcome assessment. This was a longitudinal cohort study without blinding.

Reporting for specific materials, systems and methods

We require information from authors about some types of materials, experimental systems and methods used in many studies. Here, indicate whether each material, system or method listed is relevant to your study. If you are not sure if a list item applies to your research, read the appropriate section before selecting a response.

Materials & experimental systems

n/a	Involved in the study	
☐	☒	Antibodies
☒	☐	Eukaryotic cell lines
☒	☐	Palaeontology and archaeology
☒	☐	Animals and other organisms
☐	☒	Human research participants
☐	☒	Clinical data
☒	☐	Dual use research of concern

Methods

n/a	Involved in the study	
☒	☐	ChIP-seq
☒	☐	Flow cytometry
☒	☐	MRI-based neuroimaging

Antibodies

Antibodies used	AR - Cell Signaling Technologies, rabbit monoclonal clone D6F11, catalog number 5153T, IF dilution 1:100<br>INSM1 - Santa Cruz Biotechnology, mouse monoclonal clone A-8, catalog number sc-271408, IF dilution 1:50<br>NKX3.1 - ThermoFisher/Biocare, rabbit polyclonal, catalog number 82788, IF dilution 1:50<br>HOXB13 - Cell Signaling Technologies, rabbit monoclonal clone D7N8O, catalog number 90944S, IF dilution 1:200<br>PowerVision Poly-HRP anti-mouse secondary antibodies - Leica, catalog number PV6114, ready to use solution (no dilution)<br>PowerVision Poly-HRP anti-rabbit secondary antibodies - Leica, catalog number PV6119, ready to use solution (no dilution)
Validation	The specificity of all antibodies was confirmed by using positive and negative control tissue samples - see supplemental figure 4.

Human research participants

Policy information about studies involving human research participants

Population characteristics	21 men ages 58-88 (median 71) from the Stand Up to Cancer Foundation/Prostate Cancer Foundation West Coast Dream Team who underwent a metastatic tumor biopsy prior to enza and a repeat biopsy at the time of progression and whose tumor cells underwent RNA-sequencing after laser capture microdissection were included.
Recruitment	Patients were recruited from 5 sites on the West Coast of North America: University of California, Davis; University of California, Los Angeles; University of California, San Francisco; Oregon Health & Sciences University; and University of British Columbia. Patients were recruited sequentially. The study was limited to those who had metastatic disease amenable to a biopsy.
Ethics oversight	University of California, Davis; University of California, Los Angeles; University of California, San Francisco; Oregon Health & Sciences University; and University of British Columbia Institutional Review Boards

Note that full information on the approval of the study protocol must also be provided in the manuscript.

Clinical data

Policy information about clinical studies

All manuscripts should comply with the ICMJEguidelines for publication of clinical research and a completedCONSORT checklist must be included with all submissions.

Clinical trial registration	NCT02432001
Study protocol	The study overview may be found here: https://clinicaltrials.gov/ct2/show/record/NCT02432001?term=NCT02432001&draw=2&rank=1
Data collection	Data were collected from the University of California, Los Angeles; University of California, Davis; University of California, San Francisco; Oregon Health & Science University; and the University of British Columbia between May 1, 2015 and October 8, 2021.
Outcomes	Primary outcome measures for the WCDT protocol: proportion of mCRPC patients with high androgen receptor activity Determined by a gene-expression-based signature for Androgen Receptor activity having a probability of >0.50. Secondary outcome measures: progression free survival and overall survival measured from the start of therapy after the baseline biopsy until progression.
